# Congenital Hyperinsulinism: Current Laboratory-Based Approaches to the Genetic Diagnosis of a Heterogeneous Disease

**DOI:** 10.3389/fendo.2022.873254

**Published:** 2022-07-07

**Authors:** Thomas I. Hewat, Matthew B. Johnson, Sarah E. Flanagan

**Affiliations:** Institute of Biomedical and Clinical Science, University of Exeter Medical School, Exeter, United Kingdom

**Keywords:** hyperinsulinism, hypoglycaemia, genetic screening, genetics, next generation sequencing - NGS

## Abstract

Congenital hyperinsulinism is characterised by the inappropriate release of insulin during hypoglycaemia. This potentially life-threatening disorder can occur in isolation, or present as a feature of syndromic disease. Establishing the underlying aetiology of the hyperinsulinism is critical for guiding medical management of this condition especially in children with diazoxide-unresponsive hyperinsulinism where the underlying genetics determines whether focal or diffuse pancreatic disease is present. Disease-causing single nucleotide variants affecting over 30 genes are known to cause persistent hyperinsulinism with mutations in the KATP channel genes (*ABCC8* and *KCNJ11*) most commonly identified in children with severe persistent disease. Defects in methylation, changes in chromosome number, and large deletions and duplications disrupting multiple genes are also well described in congenital hyperinsulinism, further highlighting the genetic heterogeneity of this condition. Next-generation sequencing has revolutionised the approach to genetic testing for congenital hyperinsulinism with targeted gene panels, exome, and genome sequencing being highly sensitive methods for the analysis of multiple disease genes in a single reaction. It should though be recognised that limitations remain with next-generation sequencing with no single application able to detect all reported forms of genetic variation. This is an important consideration for hyperinsulinism genetic testing as comprehensive screening may require multiple investigations.

## Introduction

Persistent congenital hyperinsulinism (HI) is characterised by the inappropriate secretion of insulin during hypoglycaemia which continues beyond 3 months. A prompt diagnosis of HI and effective management of glucose levels is critical to prevent adverse outcomes (1).

Persistent HI affects approximately 1 in 13,500 to 1 in 45,000 new-borns in non-consanguineous populations (2–5). In some isolated communities where founder mutations have been reported, and in populations with high rates of consanguinity, the incidence can increase to approximately 1 in 3,000 (6, 7). At least 36 different genetic causes of HI have been reported which follow recessive, dominant, X-linked, or sporadic inheritance (**Table 1**). The underlying genetic aetiology will determine whether the HI presents as isolated pancreatic disease or occurs as part of a rare syndrome.

**Table 1:** Known genetic causes of isolated congenital hyperinsulinism and current approaches to genetic testing for this condition. A tick (✓) or cross (X) denote whether the form of genetic variation can be detected by the screening approach. None of the variants listed will be detected by methylation studies or array-CGH analysis. SNVs are single nucleotide variants, Indels are insertion/deletion variants and CNVs are copy number variants (deletions and duplications).

Gene	Zygosity	Mutation type	SangerSequencing^1^	Next Generation Sequencing			Ref
				Targeted Panel	Exome	Genome	
*ABCC8*	Dominant or recessive	SNVs/indels	✓	✓	✓^2^	✓	(8–10)
		Large CNVs	X	✓	✓	✓
*GCK*	Dominant	SNVs/indels	✓	✓	✓	✓	(11)
*GLUD1*	Dominant	SNVs/indels	✓	✓	✓	✓	(12)
*HADH*	Recessive	SNVs/indels	✓	✓	✓^2^	✓	(13)
		Large CNVs	X	✓	✓	✓	(14)
*HK1*	Dominant	SNVs/indels	✓	✓	X	✓	(15)
		Large CNVs	X	✓	X	✓
*HNF1A*	Dominant	SNVs/indels	✓	✓	✓	✓	(16)
*HNF4A*	Dominant	SNVs/indels	✓	✓	✓^2^	✓	(17)
		Large CNVs	X	✓	✓	✓	(18)
*INSR*	Dominant	SNVs/indels	✓	✓	✓	✓	(19)
*KCNJ11*	Dominant or recessive	SNVs/indels	✓	✓	✓	✓	(20)
*SLC16A1*	Dominant	SNVs/indels	✓	✓	X	✓	(21)

**^1^** Sanger sequencing will not detect heterozygous deletions of duplications that extend beyond the targeted region. Homozygous deletions that encompass a primer binding site may be detected by a failure to amplify the sequence, but this will require verification by an independent method.

**^2^** Exome sequencing will not detect the deep intronic mutations or promoter mutations reported in these genes (22).

Many laboratories provide genetic testing for congenital HI; however, strategies vary between testing centres both in terms of the genes that are screened and the types of variation that can be detected (23–25). The different approaches to testing employed by each laboratory could help explain the differences in the percentage of mutation positive cases between cohorts which range from 45% to 79% (3, 4, 26, 27). Furthermore, the large number of genes which cause HI, the variable penetrance observed both within and between families with the same disease-causing variants, and the multiple modes of inheritance reported can hinder genetic interpretation which will also impact on the pick-up rates reported by each laboratory.

In this review, we describe the genetic causes of HI and discuss the benefits and limitations of the different methodological approaches currently used for genetic screening of this condition.

## Genetic Types of Congenital Hyperinsulinism

Disease-causing variants in 10 genes have been reported to cause isolated, persistent HI (**Table 1**). Loss-of-function variants in the *ABCC8* and *KCNJ11* genes, which encode the two subunits of the pancreatic beta-cell ATP-sensitive potassium (KATP) channel, are most common and reported in 30-66% of cases referred for genetic testing (3, 4, 26, 27). A wide range of clinical severity is associated with KATP-HI with the functionally mildest variants causing transient disease which responds well to diazoxide treatment (the frontline drug for HI), whilst the most functionally severe variants cause diazoxide-unresponsive HI that persists throughout childhood (8, 28, 29). For individuals with diazoxide-unresponsive HI, pancreatic resection may be required to prevent life-threatening hypoglycaemia. For these infants, rapid genetic testing of the KATP channel genes is critical as it will determine the histological subtype of disease. Identifying biallelic (two disease-causing variants on opposite alleles) or a single dominant KATP channel disease-causing variant confirms diffuse pancreatic disease. In contrast finding a paternally inherited, recessive KATP channel variant, predicts focal disease with a sensitivity of 97% (27, 30). In these individuals the variant is rendered homozygous by a second somatic genetic event within the pancreas (uniparental isodisomy) (31, 32). This can be genetically confirmed by testing the pancreatic tissue following a lesionectomy, which proves curative in most cases.

Clinical characteristics can help to predict some genetic forms of isolated HI. For example, high ammonia concentrations are a consistent feature of *GLUD1*-HI (12), a family history of Maturity-Onset Diabetes of the Young (MODY) can predict *HNF4A* or *HNF1A*- HI (16, 17), and exercise-induced HI suggests a role for the beta-cell disallowed gene, *SLC16A1* in disease pathogenesis (21).

Over 28 different syndromes which feature HI have been reported with the most common being Beckwith-Wiedemann syndrome (BWS) and Kabuki syndrome (33) (**Table 2**). The proportion of individuals with syndromic disease who present with HI varies between genetic subgroups. In some conditions HI is reported as a cardinal feature [e.g. Beckwith-Wiedemann syndrome (66)] whilst for others it is reported as a rare feature of the disease [e.g. Chromosome 9p deletions (40)]. Without genetic testing it can be hard to accurately diagnose syndromic disease, especially when HI is the presenting feature and dysmorphisms develop after birth, or when the clinical features are not specific to a genetic syndrome (67). For individuals with syndromic HI a genetic diagnosis is important as it will inform on prognosis and allow for the effective monitoring of new features of the disease.

**Table 2:** Known genetic causes of syndromic disease in which congenital hyperinsulinism can be a rare or common feature and the current approaches to genetic testing for this condition. A tick (✓) or cross (X) denote whether the form of genetic variation can be detected by the screening approach. Methylation studies refer to methodologies that can detect changes in DNA methylation patterns (e.g. Epic array analysis, Methylation-specific MLPA). SNVs are single nucleotide variants, Indels are insertion/deletion variants and CNVs are copy number variants (deletions and duplications).

Gene	Zygosity	Syndrome	Mutation type	SangerSequencing^1^	Next Generation Sequencing			Array- CGH	Methylation studies	Ref
					TargetedPanel	Exome	Genome			
*ABCC8*	Recessive	Usher Syndrome	Large CNVs^2^	x	✓	✓	✓	X	X	(34)
*ADK*	Recessive	*ADK* deficiency	SNVs/indels	✓	✓	✓	✓	X	X	(35)
*ALG3*	Recessive	Congenital disorder of glycosylation	SNVs/indels	✓	✓	✓	✓	X	X	(36)
*CACNA1D*	Dominant	Primary aldosteronism, seizures & neurological abnormalities	SNVs/indels	✓	✓	✓	✓	X	X	(37)
*CDKN1C*	Dominant	Beckwith-Wiedemann	SNVs/indels	✓	✓	✓	✓	X	X	(38)
Chr5q35 deletion	Dominant	Sotos	Large CNVs	X	✓	✓	✓	✓	X	(39)
Chr9p deletion	Dominant	Chr9p deletion	Large CNVs	X	✓	✓	✓	✓	X	(40)
Chr11p15.5 loss of methylation	Dominant	Beckwith-Wiedemann	Imprinting abnormality	X	X^3^	X	X	X^3^	✓	(41)
*CREBBP*	Dominant	Rubinstein-Taybi	SNVs/indels	✓	✓	✓	✓	X	X	(42)
			Large CNVs	X	✓	✓	✓	X	X
*DIS3L2*	Recessive	Perlman	SNVs/indels	✓	✓	✓	✓	X	X	(43, 44)
			Large CNVs	X	✓	✓	✓	X	X
*EIF2S3*	X-linked recessive	MEHMO	SNVs/indels	✓	✓	✓	✓	X	X	(45)
*EP300*	Dominant	Rubinstein-Taybi	SNVs/indels	✓	✓	✓	✓	X	X	(42)
			Large CNVs	X	✓	✓	✓	X	X
*FAH*	Recessive	Tyrosinaemia type I	SNVs/indels	✓	✓	✓	✓	X	X	(46)
*FOXA2*	Dominant	Syndromic	SNVs/indels	✓	✓	✓	✓	X	X	(47)
*GPC3*	X-linked recessive	Simpson-Golabi-Behmel	SNVs/indels	✓	✓	✓	✓	X	X	(48)
			Large CNVs	X	✓	✓	✓	X	X
*HNF4A*	Dominant	Fanconi renotubular syndrome 4	SNV	✓	✓	✓	✓	X	X	(16)
*HRAS*	Dominant	Costello	SNVs/indels	✓	✓	✓	✓	X	X	(49)
*KDM6A*	X-linked dominant	Kabuki	SNVs/indels	✓	✓	✓	✓	X	X	(50)
			Large CNVs	X	✓	✓	✓	X	X
*KMT2D*	Dominant	Kabuki	SNVs/indels	✓	✓	✓	✓	X	X	(51, 52)
			Large CNVs	X	✓	✓	✓	X	X
*MAGEL2*	Dominant^4^	Schaaf-Yang	SNVs/indels	✓	✓	✓	✓	X	X	(53)
*MPI*	Recessive	Congenital disorder of glycosylation	SNVs/indels	✓	✓	✓	✓	X	X	(54)
*NSD1*	Dominant	Sotos	SNVs/indels	✓	✓	✓^5^	✓	X	X	(55–57)
			Large CNVs	X	✓	✓^5^	✓	X	X
*PHOX2B*	Dominant	Congenital central hypoventilation	SNVs/indels	✓	✓	✓	✓	X	X	(58)
*PMM2*	Recessive	Polycystic Kidney Disease with HI	SNVs/indels	✓	✓	X	✓	X	X	(59)
		Congenital disorder of glycosylation	SNVs/indels	✓	✓	✓	✓	X	X	(60)
Trisomy 13	Dominant	Patau	Aneuploidy (Trisomy)	X	✓	✓	✓	✓	X	(61)
*TRMT10A*	Recessive	Syndromic	SNVs/indels	✓	✓	✓	✓	X	X	(62)
*YARS*	Recessive	Syndromic	SNVs/indels	✓	✓	✓	✓	X	X	(63)
45,X	Dominant	Turner	Aneuploidy (Monosomy)	X	✓	✓	✓	✓	X	(64)

**^1^** Sanger sequencing will not detect heterozygous deletions of duplications that extend beyond the targeted region. Homozygous deletions that encompass a primer binding site may be detected by a failure to amplify the sequence, but this will require verification by an independent method.

**^2^** Congenital hyperinsulinism, profound congenital sensorineural deafness, enteropathy and renal tubular dysfunction is causes by a contiguous deletion extending over ABCC8 and USH1C.

**^3^** Rare deletions and duplications of the Chr11p15.5 imprinted region(s) can cause Beckwith Wiedemann syndrome (65). Their size and location will determine whether they can be detected by next-generation sequencing or microarray analysis.

**^4^** MAGEL2 is an imprinted gene, loss-of-function mutations only cause disease when present on the paternal allele.

**^5^** Intergenic mutations affecting NSD1 have been reported; these would not be detected by exome sequencing (55).

## Sanger Sequencing

Causative genes for HI were historically screened by Sanger sequencing; an approach that allows a few hundred nucleotides (typically a single exon) to be rapidly sequenced in a single reaction. This is followed by semi-automated analysis by alignment and inspection of the DNA sequence. These constraints force laboratories to screen genes sequentially in descending order of prior probability based on clinical characteristics and how commonly disease-causing variants in the gene are identified. Whilst this phenotype-driven approach works well in many scenarios [for example in the rapid screening of KATP channel genes in individuals with diazoxide-unresponsive disease (68, 69)], the reliance of clinical features to guide testing can delay a genetic diagnosis for individuals with an atypical presentation. This is an important consideration for HI, as phenotypic variability is described within most genetic subgroups, for example the presence of normal ammonia levels in some children with *GLUD1*-HI (70, 71). Using the clinical characteristics to guide genetic testing in syndromic HI should also be applied with caution as additional features may develop after the diagnosis of HI (72).

A further major limitation of Sanger sequencing is its inability to detect heterozygous deletions and duplications that extend beyond the targeted region, changes in the number of chromosomes (aneuploidies), and defects in methylation, all of which have been reported to cause HI (**Table 1**).

Despite its limitations, Sanger sequencing remains a highly sensitive test for the rapid detection of single-nucleotide variants and small insertion/deletion variants (indels) in both the coding and non-coding regions of the genome. It can also detect mosaic variants (i.e. a genetic variant that is introduced during cell division that does not affect every cell within the body) that are present in the sampled tissue at a level of >8% (73). This is important, as disease-causing mosaic variants have been reported in the known HI genes including *KMT2D*, *KDM6A*, *NSD1*, and *CREBBP* (74–76).

## Next-Generation Sequencing

Since 2005, next-generation sequencing has provided a method to allow for the simultaneous analysis of multiple genes in a single assay (77). This technology revolutionised diagnostic testing for genetically heterogeneous disorders such as HI by allowing for the parallel screening of all known disease-causing genes/genomic regions in a single assay at a much lower cost than Sanger sequencing. This led to a paradigm shift for conditions like syndromic HI where genetic testing can precede the development of the full clinical spectrum of disease, serving to make, rather than confirm, the clinical diagnosis (67).

### Targeted Gene Panel Analysis by Next-Generation Sequencing

A targeted gene panel typically includes all known genetic causes of a disease and DNA samples are enriched for DNA in these loci prior to next-generation sequencing. For most targeted gene panels, the average coverage achieved often reaches many hundreds of reads over each base (78). This high-depth sequencing data can be exploited to detect changes in copy number over targeted regions and allows for the accurate detection of mosaic variants occurring at a level of >1% (73). Recent studies have shown that off-target reads generated during the sequencing process can be analysed to assess read-depth across the entire genome allowing for the detection of large deletions and duplications outside of targeted regions (79). These off-target reads have been used successfully to detect disease-causing deletions on chromosome 9p in individuals with HI (40). The potential to identify large deletions and duplications from off-target reads will though depend on the methodology used for the targeted next-generation sequencing; amplicon-based approaches that sequence PCR products will not generate the off-target sequencing data.

The major limitation of targeted next-generation sequencing is that it only allows screening of a predetermined list of genomic regions, and this list often differs between laboratories. For genetically heterogenous conditions such as HI, it is therefore important that clinicians who order panel testing are aware of which genes are included on the targeted panels and whether copy number analysis has been performed as this requires a separate bioinformatic analysis.

### Exome and Genome Sequencing

The introduction of next-generation sequencing has enabled the rapid sequencing of the coding regions of all genes (the exome) or the entire human genome (coding and non-coding regions) at much lower cost than previous methods. The approach to the interpretation of exome and genome sequencing data will differ between centres with some analysing variants called within a pre-defined set of known disease-causing genes whilst other laboratories will perform a gene-agnostic analysis. The latter approach has the advantage of being able to identify new genes for HI, with recent successes including the discovery of the syndromic HI genes *CACNA1D*, *PMM2*, *FOXA2*, *TRMT10A*, *EIF2S3*, *YARS*, and *KMT2D* by exome sequencing and more recently the finding of regulatory variants deep within intron 2 of the beta-cell disallowed gene, *HK1*, by genome sequencing in individuals with isolated hyperinsulinism (15, 37, 45, 47, 51, 59, 62, 63). The ability of a laboratory to utilise next-generation sequencing data for genetic discovery will largely depend on their ability to perform robust genetic and functional studies to assess novel variation.

Exome sequencing targets the ~2% of the genome which codes for protein, making it a cheaper alternative to genome sequencing. This, together with the knowledge that 85% of known disease-causing mutations reside within coding regions, has led to exome sequencing being widely adopted within the clinical setting (80). For example, in the UK, rapid exome sequencing for acutely unwell neonates is available through the country’s National Health Service with 38% of patients tested receiving a rapid diagnosis (81). Unlike targeted next-generation sequencing, which screens a predetermined list of genes, exome sequencing provides an extremely effective method to comprehensively analyse the coding regions and intron/exon boundaries of all known HI genes and to assess copy number status. The major limitation of the approach is that it will not detect non-coding mutations such as the deep intronic mutations reported in *ABCC8*, *HADH* and *HK1* or promoter variants in genes such as *HNF4A*, *PMM2*, and *SLC16A1* (15, 21, 22, 59, 82).

Genome sequencing represents the gold standard approach to genetic testing given its ability to detect the largest range of genetic variation. As well as providing data on coding and non-coding regions, genome sequencing can be used to search for structural changes, copy number variants (large deletions, duplications, and aneuploidies) and mosaic variants although the lower read depth achieved makes this a less sensitive approach for detecting low-level mosaic variants compared to targeted next-generation sequencing.

The costs associated with sequencing the entire genome and the large amount of data produced (approximately 200GB of processed data per sample versus 11GB per sample for exome sequencing) had prohibited the adoption of routine genome sequencing. Until recently it had been largely reserved for genetic screening when a disease-causing variant had not been detected by targeted next-generation sequencing or exome sequencing. This approach successful resulted in an increase in diagnostic yield for many rare genetic diseases (83, 84).

Improvements in sequencing capabilities leading to reduced costs are though now leading to the emergence of genome sequencing as a first line diagnostic test in specific healthcare settings, for example in the screening of some rare developmental disorders in the UK National Health Service (85). While genome sequencing is not the current approach for investigating the genetic cause of HI in many centres, it seems likely that this will become the first line test in the coming years.

## Non Sequencing Based Methods to Detect Copy Number Variants and Methylation Defects

Aneuploidies and large deletions and duplications (copy number variants) are a rare but important cause of HI (**Tables 1** and **2**). Unlike Sanger sequencing, next-generation sequencing can detect these forms of genetic variation, but many laboratories will not routinely screen for them as a separate analysis pipeline is required. This is an important consideration when disease-causing variants are not detected in children with HI and particularly for those where there are additional syndromic features (**Table 2**).

Multiplex-ligation dependent probe amplification (MLPA) can detect disease-causing deletions and duplications in individuals with HI. This approach is commonly used to screen for deletions in the *ABCC8* gene and can detect mosaicism (9). The usefulness of MLPA is limited by its ability to analyse a maximum of 60 different small genomic regions (generally single exons) in a single assay thus preventing the simultaneous analysis of all HI genes in which copy number changes have been reported.

Microarray-based comparative genomic hybridization (array CGH) is a well-established method that is used to detect large deletions/duplications and aneuploidies in individuals with HI. Unlike MLPA, array GCH is not able to detect low level mosaicism (<30% mosaicism for deletions and duplications and <10% for aneuploidies). The approach does however allow for the analysis of copy number variation across a greater percentage of the genome although the targeted region will vary across arrays and will not always target the regions known to cause HI with enough precision.

Current diagnostic sequencing approaches are also unable to detect changes in DNA methylation. Individuals with clinical suspicion of an imprinting disorder such as Beckwith-Wiedemann syndrome may therefore require additional methylation studies, such as methylation-specific MLPA (MS-MLPA) (86) or Infinium Methylation EPIC array analysis (87). Emerging technologies, such as Oxford Nanopore sequencing, may allow for the simultaneous detection of sequence variation and DNA methylation status but have not been widely used clinically. This technology does offer the hope of a single comprehensive test for genetically heterogeneous disorders like HI although to date it has mainly been used for genes that are hard to sequence by other methodologies (88–91).

## Further Considerations and Concluding Remarks

Diagnostic testing for HI is routinely performed on DNA extracted from peripheral blood leukocytes, saliva, or buccal samples. For conditions such as HI it is important to consider the source of DNA being screened, given that somatic mutations which are only present in the pancreatic tissue have been reported (27, 92). Therefore, when a mutation is not identified in the blood, and a pancreatectomy has been performed, re-testing the known HI genes to search for a variant present only within the pancreatic DNA should be considered.

In conclusion, several different genetic approaches exist for routine diagnostic screening in HI with genome sequencing representing the gold standard approach to testing. For healthcare professionals managing this genetically heterogenous disorder it is important that the limitations of each approach including genome sequencing, are recognised as no single test can detect all known types of genetic variation reported in HI. This is particularly important when managing syndromic disease, where copy number variants or defects in methylation are common. Despite there being a broad range of genetic screening approaches that are available for HI, in reality the testing strategy is most likely to be influenced by the capabilities of the local genetic diagnostic laboratory, affordability and importantly how quickly the tests can be performed and results reported back. This is especially critical for children with diazoxide-unresponsive disease as identifying a paternally inherited KATP disease-causing variant suggests focal pancreatic disease which can be cured by lesionectomy.

## Author Contributions

TH and SF performed the literature searches and reviews. TH, MJ, and SF drafted and reviewed the manuscript. All authors contributed to the article and approved the submitted version.

## Funding

SF is a Wellcome Trust Senior Research Fellow (Grant Number 223187/Z/21/Z). TH is the recipient of a PhD studentship and MJ has an Independent Fellowship from the Exeter Diabetes Centre of Excellence funded by Research England’s Expanding Excellence in England (E3) fund.

## Conflict of Interest

The authors declare that the research was conducted in the absence of any commercial or financial relationships that could be construed as a potential conflict of interest.

## Publisher’s Note

All claims expressed in this article are solely those of the authors and do not necessarily represent those of their affiliated organizations, or those of the publisher, the editors and the reviewers. Any product that may be evaluated in this article, or claim that may be made by its manufacturer, is not guaranteed or endorsed by the publisher.
